# Community-Based Digital Contact Tracing of Emerging Infectious Diseases: Design and Implementation Study With Empirical COVID-19 Cases

**DOI:** 10.2196/47219

**Published:** 2023-11-08

**Authors:** Hsiao-Chi Wang, Ting-Yu Lin, Yu-Chin Yao, Chen-Yang Hsu, Chang-Jung Yang, Tony Hsiu-Hsi Chen, Yen-Po Yeh

**Affiliations:** 1 Changhua County Public Health Bureau Changhua County Taiwan; 2 Institute of Epidemiology and Preventive Medicine College of Public Health National Taiwan University Taipei Taiwan; 3 Master of Public Health Program College of Public Health National Taiwan University Taipei Taiwan

**Keywords:** COVID-19, digital contact tracing, public health, surveillance

## Abstract

**Background:**

Contact tracing for containing emerging infectious diseases such as COVID-19 is resource intensive and requires digital transformation to enable timely decision-making.

**Objective:**

This study demonstrates the design and implementation of digital contact tracing using multimodal health informatics to efficiently collect personal information and contain community outbreaks. The implementation of digital contact tracing was further illustrated by 3 empirical SARS-CoV-2 infection clusters.

**Methods:**

The implementation in Changhua, Taiwan, served as a demonstration of the multisectoral informatics and connectivity between electronic health systems needed for digital contact tracing. The framework incorporates traditional travel, occupation, contact, and cluster approaches and a dynamic contact process enabled by digital technology. A centralized registry system, accessible only to authorized health personnel, ensures privacy and data security. The efficiency of the digital contact tracing system was evaluated through a field study in Changhua.

**Results:**

The digital contact tracing system integrates the immigration registry, communicable disease report system, and national health records to provide real-time information about travel, occupation, contact, and clusters for potential contacts and to facilitate a timely assessment of the risk of COVID-19 transmission. The digitalized system allows for informed decision-making regarding quarantine, isolation, and treatment, with a focus on personal privacy. In the first cluster infection, the system monitored 665 contacts and isolated 4 (0.6%) cases; none of the contacts (0/665, 0%) were infected during quarantine. The estimated reproduction number of 0.92 suggests an effective containment strategy for preventing community-acquired outbreak. The system was also used in a cluster investigation involving foreign workers, where none of the 462 contacts (0/462, 0%) tested positive for SARS-CoV-2.

**Conclusions:**

By integrating the multisectoral database, the contact tracing process can be digitalized to provide the information required for risk assessment and decision-making in a timely manner to contain a community-acquired outbreak when facing the outbreak of emerging infectious disease.

## Introduction

### Rationale of Contact Tracing for Containing Infectious Diseases

Contact tracing, a key element of nonpharmaceutical interventions (NPIs), has been demonstrated as an effective approach in reducing the spread of emerging infectious diseases (EIDs), such as COVID-19 in the early phase of the global pandemic in 2020, when effective vaccines and antiviral therapies were not yet available [1,2]. Individuals who are more likely to contract SARS-CoV-2 can be identified through contact tracing. Supported by this knowledge, measures such as quarantine, isolation, symptom monitoring, and sanitary practices can be applied to forestall a community-acquired epidemic [3-13]. However, acquiring the information required for precise NPIs through traditional contact tracing is a time-consuming and labor-intensive technique that may impede the timely implementation of containment measures.

### The Needs of Digital Contact Tracing

As an efficient alternative, digital contact tracing uses big data analytics to locate all potential contacts by integrating accessible information. With this detailed knowledge, the two factors, contact rate and transmission probability, embedded in the effective reproductive number can be minimized to forestall the outbreak of an EID such as COVID-19 in a community. Since the beginning of the COVID-19 pandemic, digital contact tracing has been proposed and implemented in a variety of nations and regions [5,8,10,11,13-23]. The implementation of contact tracing in the context of containment measures may vary from time to time, not only due to the transmissibility and severity of dominant COVID-19 variants of concern (VOCs) but also the availability of vaccines and antiviral therapies [24,25]. Nevertheless, there have been few attempts to examine the details of implementations of digital contact tracing based on empirical cases of efforts to contain COVID-19 outbreaks [14,16,18,19,22].

### Digital Contact Tracing in Taiwan

Taiwan implemented digital contact tracing based on multiple information sources during the early phases of the COVID-19 pandemic. Before the occurrence of community-acquired clusters, digital methods were used to trace potential contacts from the *Diamond Princess* cruise ship [5]. These digital techniques included sensor data, such as GPS information from shuttle buses, highway electronic toll collection systems, credit card transaction logs, closed-circuit television records, and mobile position data, for tracing and providing warning messages to potential contacts [5]. Supported by the Taiwan Communicable Disease Control Act [26], consent for the retrieval of individual information related to the containment of EID outbreaks under government auspices was waived. Taiwan’s experience in containing the severe acute respiratory syndrome outbreak in 2003 and the early adoption of digital contact tracing during the COVID-19 pandemic, as justified by the Taiwan Communicable Disease Control Act, have established a strong foundation for the mandatory provision of individual data to the government with the aim of containing EIDs and preserving collective benefits. This acceptance within the Taiwanese population also provides a solid basis for the evolution of digital contact tracing.

Since the initial identification of community-acquired transmission of COVID-19 in January 2020 in Changhua, Taiwan, a series of contact tracing measures have been implemented to mitigate the spread of the virus in the community. To ensure the efficient deployment of NPIs, the Changhua Health Administration (Changhua County Public Health Bureau) has adopted digitally transformed approaches since early February 2020. This study aimed to demonstrate the design and implementation of digital contact tracing. The evolution of this digital cluster investigation was further illustrated by 3 empirical SARS-CoV-2 infection clusters in Changhua. The effectiveness in containing a community-acquired outbreak by using digital contact tracing was evaluated by using effective reproductive number as an indicator.

## Methods

### Overview

On January 20, 2020, the first domestic case, which was also the first community-acquired transmission of COVID-19 in Taiwan, occurred in Changhua. This COVID-19 cluster involved an index case identified through symptom-based surveillance for COVID-19 and a household member of this index case [27]. Following this household transmission, the Changhua County Public Health Bureau switched from traditional contact tracing to a digital strategy to effectively contain the spread from household to community. The framework and the implementation of this digital transformation are described in the following sections.

### Big Data Integration for Contact Tracing

#### The Framework

**Figure 1:**
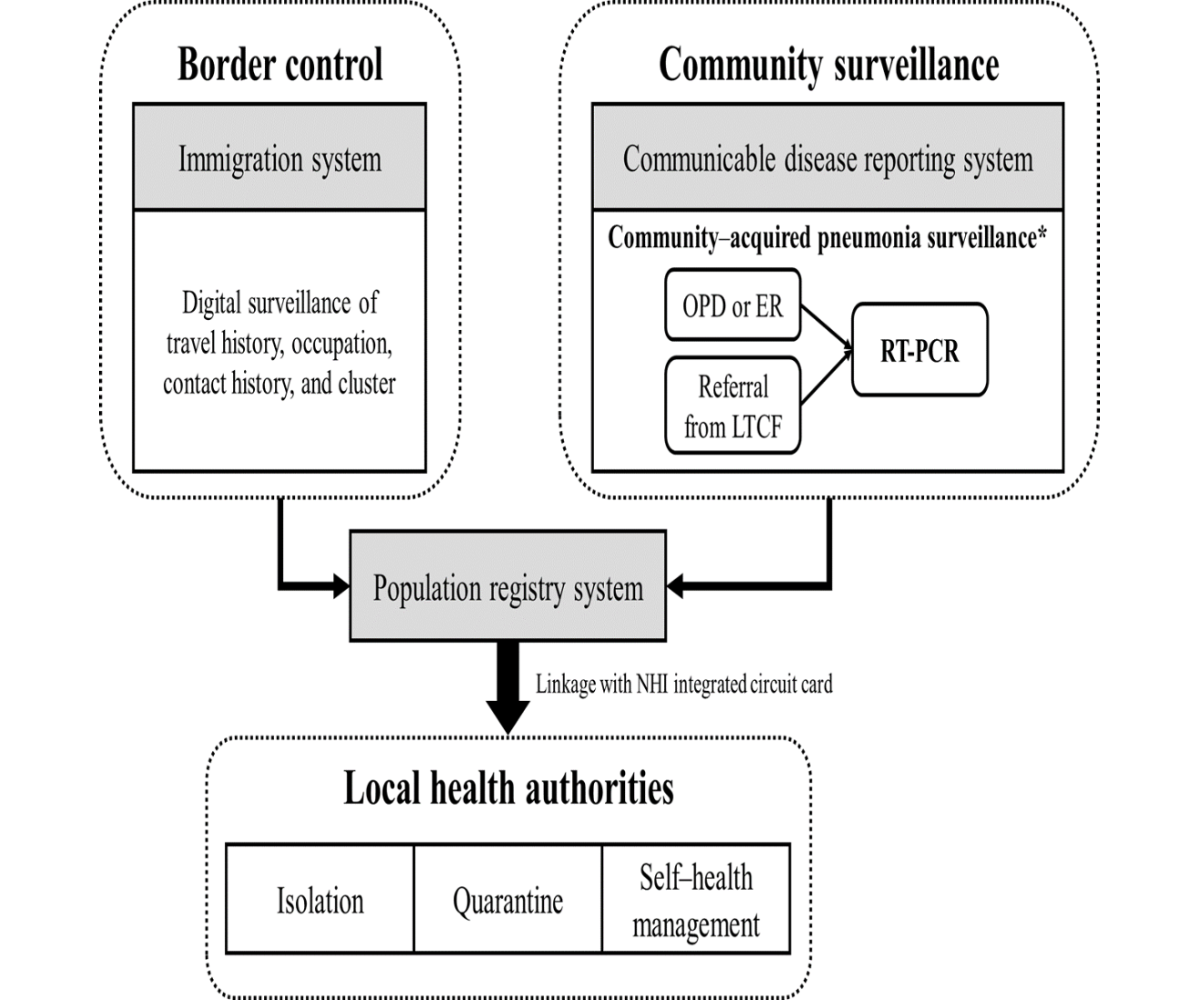
Illustration of immigration data linkage with NHI claim data and the corresponding preventive measures. *Patients in the OPD, ER, or LTCFs. RT-PCR: ER: emergency room; LTCF: long-term care facility; NHI: National Health Insurance; OPD: outpatient department; RT-PCR: reverse transcriptase polymerase chain reaction.

Figure 1 shows the configuration of the integrated database, which is of cardinal importance for digital contact tracing. The integration of big data for digital contact tracing was facilitated by the Taiwan National Health Insurance (NHI) digital health care system, which covers 99% of the population [28,29]. The auxiliary databases including the community surveillance system for infectious disease (communicable disease reporting registry), border control system (immigration registry), and population residence registry (registry of civil affairs) were incorporated into the NHI registry. The following steps are outlined for combining these multidimensional databases from multiple sources to create an integrated digital platform for contact tracing.

#### Step 1: Immigration Database and Community Surveillance Interlinked With NHI Health Care Registry

##### Overview

As depicted in Figure 1, the information system needed for contact tracing consists of 2 registries, the communicable disease reporting system for community monitoring and the immigration system for border control. The specification of immigration history and medical history of reportable communicable diseases at the individual level can be considered a legitimate response to the emerging COVID-19 outbreak, as supported by the Communicable Disease Control Act amended in 2019 [26], as the continuation of infrastructure renewal in response to the severe acute respiratory syndrome outbreak in 2003.

The communicable disease reporting system has detailed information about testing and health outcomes related to COVID-19 for each confirmed case, whereas the immigration registry provides information about the departing nation of an individual and the date on which the immigrant arrived in Taiwan. Major contact information, such as travel history, occupation, contact history, and cluster events, can be gathered using the personal information derived from these 2 databases.

**Figure 2:**
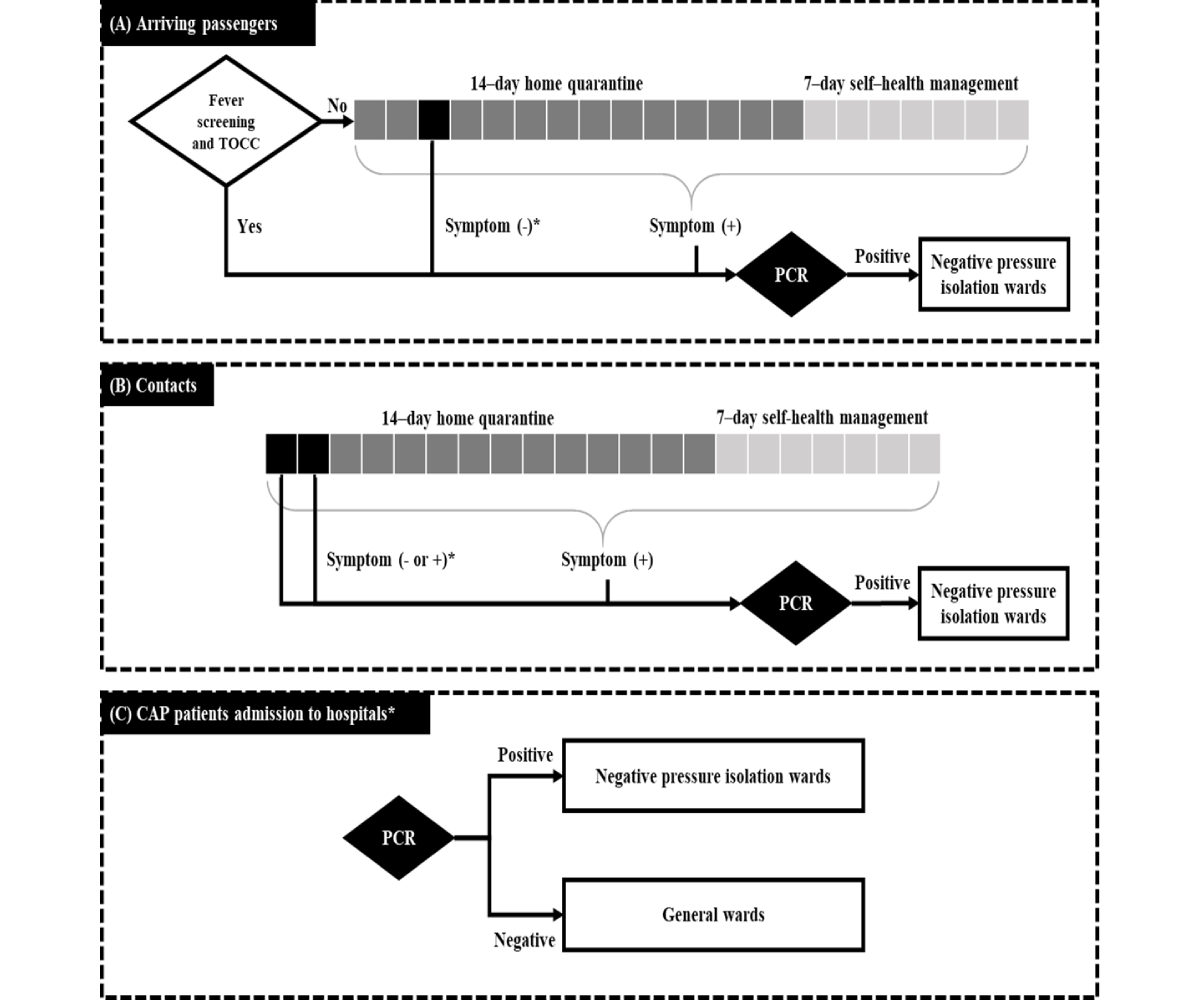
Surveillance strategies for COVID-19 in (A) arrival passengers, (B) contacts, and (C) patients with community-acquired pneumonia. *The intervention was implemented only in Changhua County. CAP: community-acquired pneumonia; PCR: polymerase chain reaction; TOCC: travel, occupation, contact, and cluster.

Overall, 3 scenarios are shown in Figure 2: arriving passengers (Figure 2A), contacts of COVID-19 cases (Figure 2B), and patients with pneumonia admitted to hospitals (Figure 2C).

##### Arriving Passengers

Before passport control, arriving travelers are required to complete a travel, occupation, contact, and cluster (TOCC) inquiry and symptom-based screening for fever, upper respiratory symptoms, gastrointestinal symptoms, anosmia, and ageusia. Passengers whose symptom-based screening findings are positive will be transferred for polymerase chain reaction testing for SARS-CoV-2 infection and further classified based on the results (Figure 2A).

Passengers without COVID-19–related symptoms are required to observe a 14-day home quarantine in a single room away from other household members. During the quarantine period, individuals are checked for COVID-19–related symptoms on the 1st, 4th, 7th, and 14th days by public health officers. To reduce the risk of transmission during the presymptomatic or asymptomatic phases, reverse transcriptase polymerase chain reaction (RT-PCR) testing is performed upon the onset of COVID-19–related symptoms or on the 3rd day for those who have not developed symptoms. After a 14-day quarantine, passengers must undergo 7 days of self–health management. Family members in the same household as the arriving passenger must also practice self–health management.

##### Contacts of COVID-19 Cases

A 14-day home quarantine is mandatory for individuals who have been in contact with cases confirmed through digital contact tracing. Public health officers conduct symptom-based surveillance similar to the process for arriving travelers. Contacts will undergo serial RT-PCR testing on the first and second days of quarantine, regardless of whether they were asymptomatic or symptomatic, and whenever COVID-19–related symptoms appear. Following the 14-day home quarantine, a 7-day period of self–health management is recommended (Figure 2B).

##### Patients With Community-Acquired Pneumonia

All hospitalized patients diagnosed with community-acquired pneumonia undergo routine RT-PCR testing to monitor the risk of COVID-19 transmission and reduce health care–associated infections. The test results will determine the need for isolation (Figure 2C).

#### Step 2: Construction of Information System for Digital Contact Tracing

Through the NHI platform, testing results, COVID-19 disease status, number of days since an international traveler arrived in Taiwan, and the postarrival quarantine destination of a traveler can be integrated to provide the essential information for digital contact tracing. Authorized individuals, such as public health officials and health care workers, can access this information after digitally confirming their identity through an integrated circuit smart card interface.

The implementation of digital contact tracing in Changhua was further enhanced through collaboration between the health administration and the police department. This collaboration included the use of closed-circuit television and geopositioning data from a network-based mobile phone tracking system. The information obtained from digital contact tracing can be seamlessly integrated with conventional approaches, including personal interviews and on-site investigations, to validate the data.

**Figure 3:**
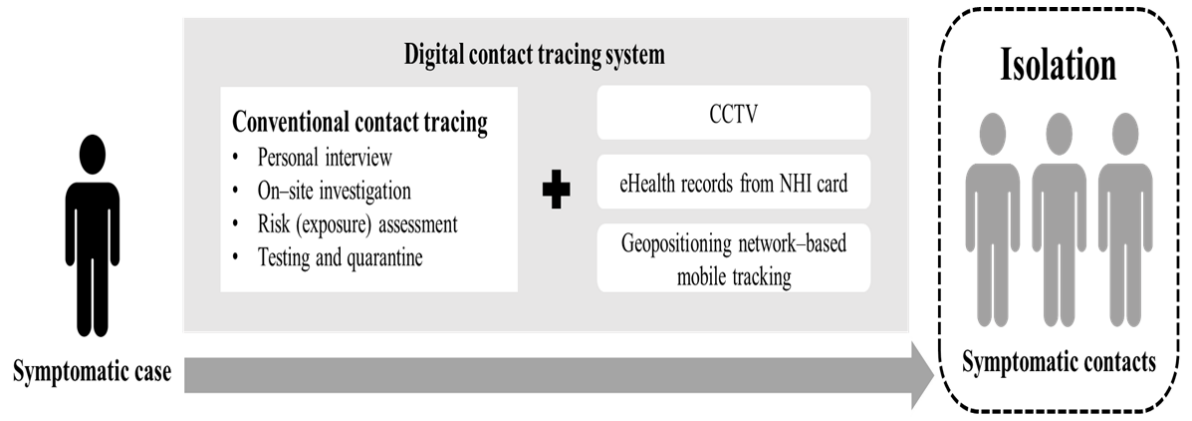
Changhua digital contact tracing system. CCTV: closed-circuit television.

Figure 3 illustrates the framework of this expanded digital contact tracing system, which was specifically tailored to meet the information gathering and risk-based action requirements for containing the COVID-19 epidemic.

As part of Taiwan’s level-3 NPI measures [30,31], it has become mandatory to collect contact information from residents entering all venues and using public transportation. Nevertheless, the process of manually completing forms was complex and presented a substantial barrier to routine practice, hindering the implementation of contact tracing strategies. To address this issue, a real-time digital registration system was developed and launched on May 19, 2021. This system uses a QR code in conjunction with an SMS text message to gather information, including phone number and time of visit, for each visitor, with a unique code attached to the visited site.

The registration process is quick and easy, consisting of three steps that can be completed in just 5 seconds: (1) scan the QR code with a mobile phone, (2) click on the link that appears, and (3) send the automatically generated SMS text message (as shown in Figure S1 in Multimedia Appendix 1). A centralized registry system maintained by the central health administration ensures the privacy and security of the collected information, eliminating the necessity of disclosing personal information. The collected information will be retained for 28 days and then erased to ensure personal privacy.

Instead of relying on a contact tracing app for collecting information about contact histories, we use the established geopositioning data from a network-based mobile phone tracking system [5] and self-reported exposure history from the QR code and SMS text message system. Given an exposure history, a warning message and the targets of contact tracing could be retrieved digitally upon the identification of a confirmed COVID-19 case. All the individuals with overlapping tracing were considered as contacts, and messages for self–health monitoring were sent. High-risk contacts, such as household members, those who had meals with the confirmed case, or long-term social contacts (eg, those residing together), were then identified by public health officials. Such an approach alleviates the issues of adaptation, updating, and user behavior (eg, turning off the contact tracing app) that may affect the effectiveness of digital contact tracing [18,32].

**Figure 4:**
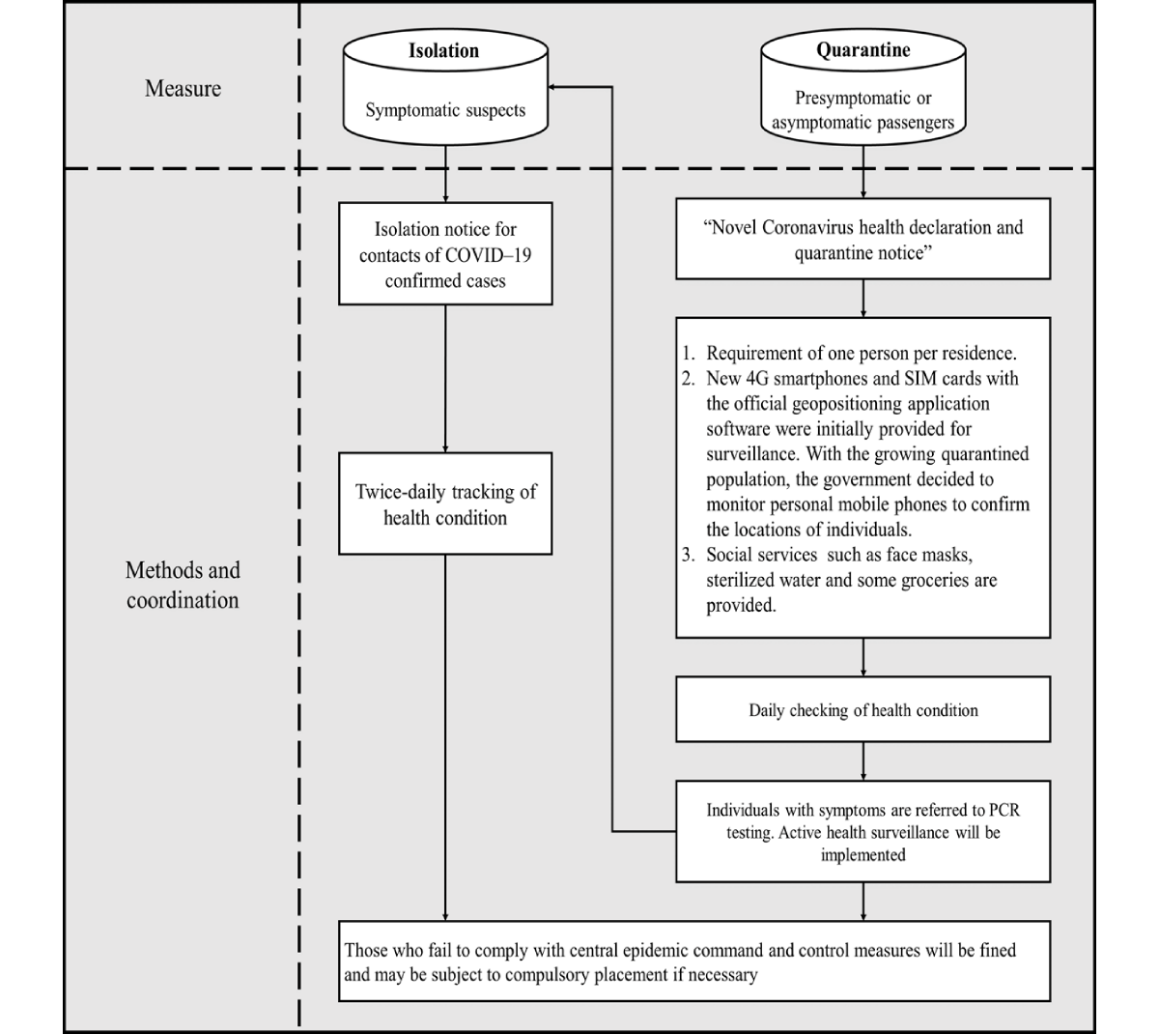
Framework of quarantine and isolation assisted by digital contact tracing. PCR: polymerase chain reaction.

This digital infrastructure provides a flexible and secure platform for public health officials and health care personnel to identify individuals at risk of SARS-CoV-2 infection. With the assistance of this integrated information system, quarantine and isolation can be precisely conducted and monitored. When residents visit a health care facility with COVID-19 symptoms, TOCC records can be retrieved using the digital identity of the health care worker and the patient’s smart card. Those with a positive history of TOCC will be scheduled for an RT-PCR test and receive 1 of 3 NPIs based on their level of exposure and testing results, including self–health management, quarantine, or isolation. In addition to routine surveillance for inbound passengers requiring quarantine and symptom-based monitoring, this integrated framework can expedite the contact tracing process initiated by public health professionals to identify COVID-19 cases. Figure 4 shows the framework for quarantine and isolation enabled by digital contact tracing technologies.

### Evaluating the Effectiveness of Epidemic Control With the Reproductive Number

To assess the effectiveness of digital contact tracing in containing the transmission of COVID-19, a comparison between the effective reproduction number (Rt) and the basic reproduction number (R0) was performed. R0 is determined by 3 factors: contact rate, transmission probability for each contact, and duration of infection, as follows:

R0 = contact per day × transmission probability of each contact × infectious period in days **(1)**

In a population that is vulnerable to SARS-CoV-2 infection, the value of R0 represents the average number of cases infected for each symptomatic case. Effective interventions can reduce each R0 factor, leading to a lower Rt value. An Rt value <1 suggests that the disease will eventually vanish without causing a large-scale community-acquired outbreak.

### Ethical Considerations

The study was approved by the Taipei Medical University joint institutional review board (N202007018). The provision of individual information, including health data, TOCC, and contact history was mandatory during the outbreak period as justified by the Taiwan Communicable Disease Control Act [26]. According to the act, consent for the retrieval of individual information related to the containment of EID outbreaks can be waived under government auspices.

## Results

We demonstrate the investigation of 3 empirical COVID-19 clusters to illustrate how digital contact tracing in Changhua shifted from a traditional approach to an IT-assisted and big data–assisted approach.

### Cluster Investigation 1 (First Household Cluster Infection in Taiwan)

Early in January 2020, the Changhua County Public Health Bureau identified a man with no previous travel history as a contact of a COVID-19 case to assess the risk of SARS-CoV-2 infection (case 2; Figure S2A in Multimedia Appendix 1). Contact tracing was initiated following the detection of an imported COVID-19 case with a travel history to Wuhan, China, who arrived in Taiwan on January 21, 2020. Figure S2A in Multimedia Appendix 1 depicts the timeline of this clustered event. When the individual (referred to as case 1 in Figure S2A in Multimedia Appendix 1) arrived in Taiwan, she was residing with a family member while under home quarantine in Changhua. Owing to fever and respiratory symptoms, on January 25, 2020, case 1 sought medical advice at Changhua Christian Hospital. Case 1 was subsequently isolated and received treatment in a negative pressure ward at Changhua Christian Hospital owing to a positive TOCC (as an international traveler from Wuhan, China; a hotspot in early 2020) history and being on the fourth day of quarantine. A positive RT-PCR result for SARS-CoV-2 was confirmed for case 1 on January 27, 2020 [27]. A household contact (case 2) was deemed to be at extremely high risk due to prolonged close contact with case 1. Despite only exhibiting very mild nonspecific respiratory symptoms, case 2 tested positive for COVID-19 on January 28, 2020, becoming the first case of household-acquired transmission in Taiwan [27].

The conventional contact tracing approach revealed that between January 21, 2020, and January 25, 2020, case 1 visited various places, including a traditional market, an electrical appliance shop, a supermarket, and a convenience store within the residential community. It is important to note that case 1 consistently wore a mask during these visits. In addition, case 2, a household contact of case 1, had visited multiple hardware stores and a department store before being confirmed as a COVID-19 case. A total of 36 contacts were identified through comparison of the contact tracing information for these 2 cases. All of these contacts were placed under quarantine and subsequently tested for COVID-19 using RT-PCR testing. Over a 14-day quarantine period, they were also monitored for COVID-19 symptoms. All contacts tested negative for COVID-19. Furthermore, thorough sanitation measures were implemented in the exposed environments and shops. These specific locations were then communicated to the community residents through mass media channels to ensure that individuals who were potentially exposed practiced self–health management.

After this household transmission of COVID-19, the Changhua County Public Health Bureau collaborated with the public health, civil affairs, and police departments to create a contact tracing system to contain the outbreak. However, owing to the extensive and multisource information required for effective contact tracing, the process is time consuming and challenging, despite the integrated efforts across sectors. Using the first COVID-19 cluster as an example, to assess the risk of disease transmission and contain an outbreak, the contact tracing process must identify potential contacts across various settings, including households and public gatherings such as supermarkets and social events. Drawing insights from the contact tracing efforts during the first community-acquired COVID-19 transmission in Changhua, a process aided by IT and the integration of big data from 3 sectors has been designed to enhance the efficiency of information gathering.

### Cluster Investigation 2 (First Community Cluster Infection in Taiwan)

The third COVID-19 case in Changhua (CHC), termed CHC3, was a man aged 62 years who was admitted to the intensive care unit on February 3, 2020, owing to rapid progression from an influenza-like illness to severe pneumonia with respiratory distress. Upon admission, a preliminary diagnosis of viral pneumonia, initially suspected to be caused by influenza, was made. Unfortunately, the disease progressed rapidly, and on February 15, 2020, when the COVID-19 RT-PCR test result returned positive, the patient passed away. CHC3 was the first COVID-19 case detected through community-acquired pneumonia surveillance in Changhua.

Upon confirming CHC3 as a COVID-19 case, the Changhua County Public Health Bureau promptly initiated contact tracing with the assistance of digital technology. Within 36 hours, the officials had identified the index case of the cluster. The tracing revealed that CHC3 had come into contact with a passenger who had visited China, Hong Kong, and Macau and was suspected of contracting the virus there. Although the passenger tested negative for COVID-19 in an RT-PCR test on February 16, 2020, a subsequent serological test for immunoglobulin G against SARS-CoV-2 returned positive, indicating that the passenger was indeed the index case of the cluster. Figure S2B in Multimedia Appendix 1 illustrates the timeline of COVID-19 transmission, reconstructed through digital contact tracing.

**Figure 5:**
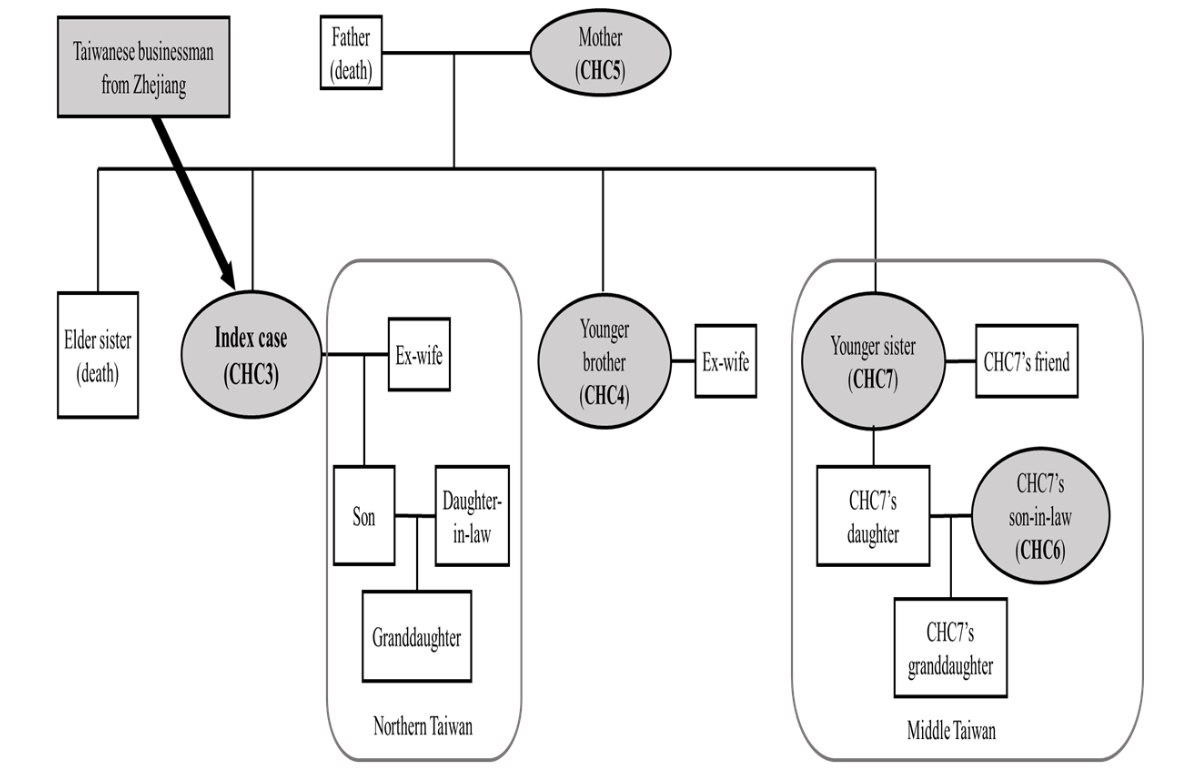
Step 1 of contact tracing for epidemic investigation in cluster 2—clarification of the family contact structure of case 3 in Changhua (CHC3). CHC: case in Changhua.

**Figure 6:**
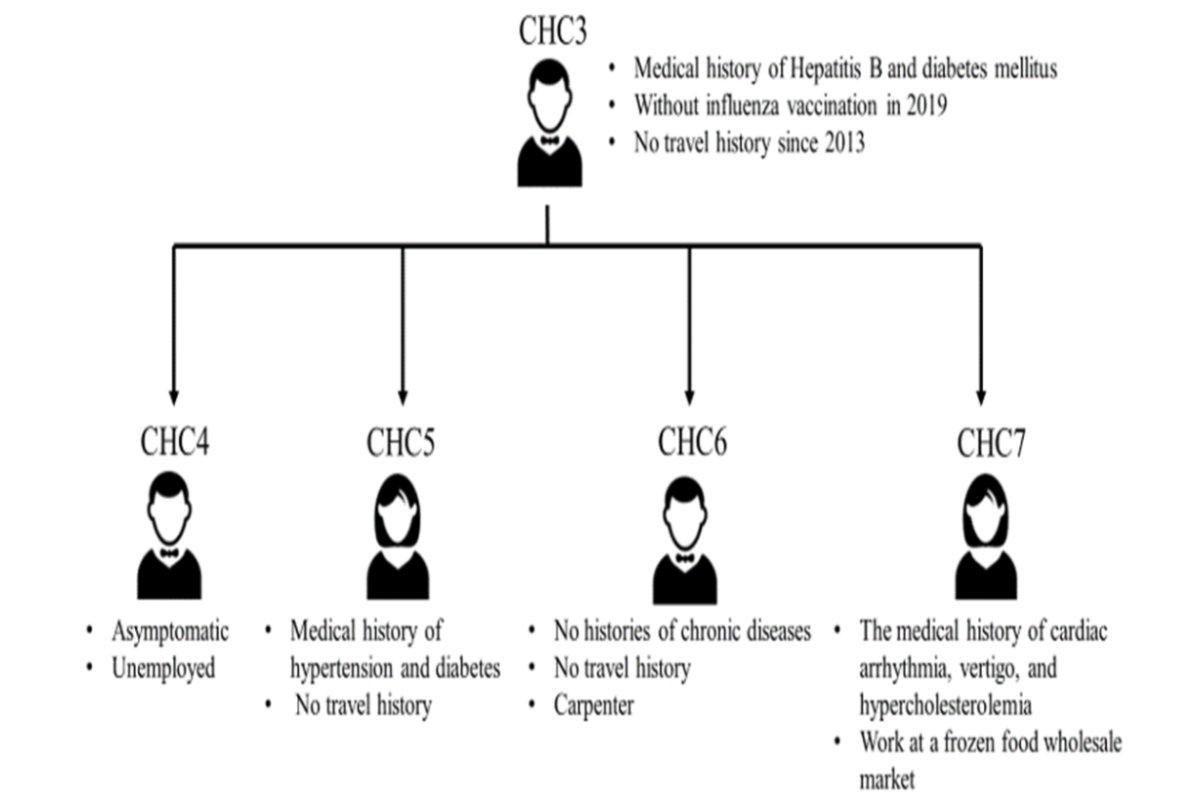
Step 2 of contact tracing of epidemic investigation in cluster 2—identifying risk information by using an integrated and digitalized information system. CHC: case in Changhua.

**Figure 7:**
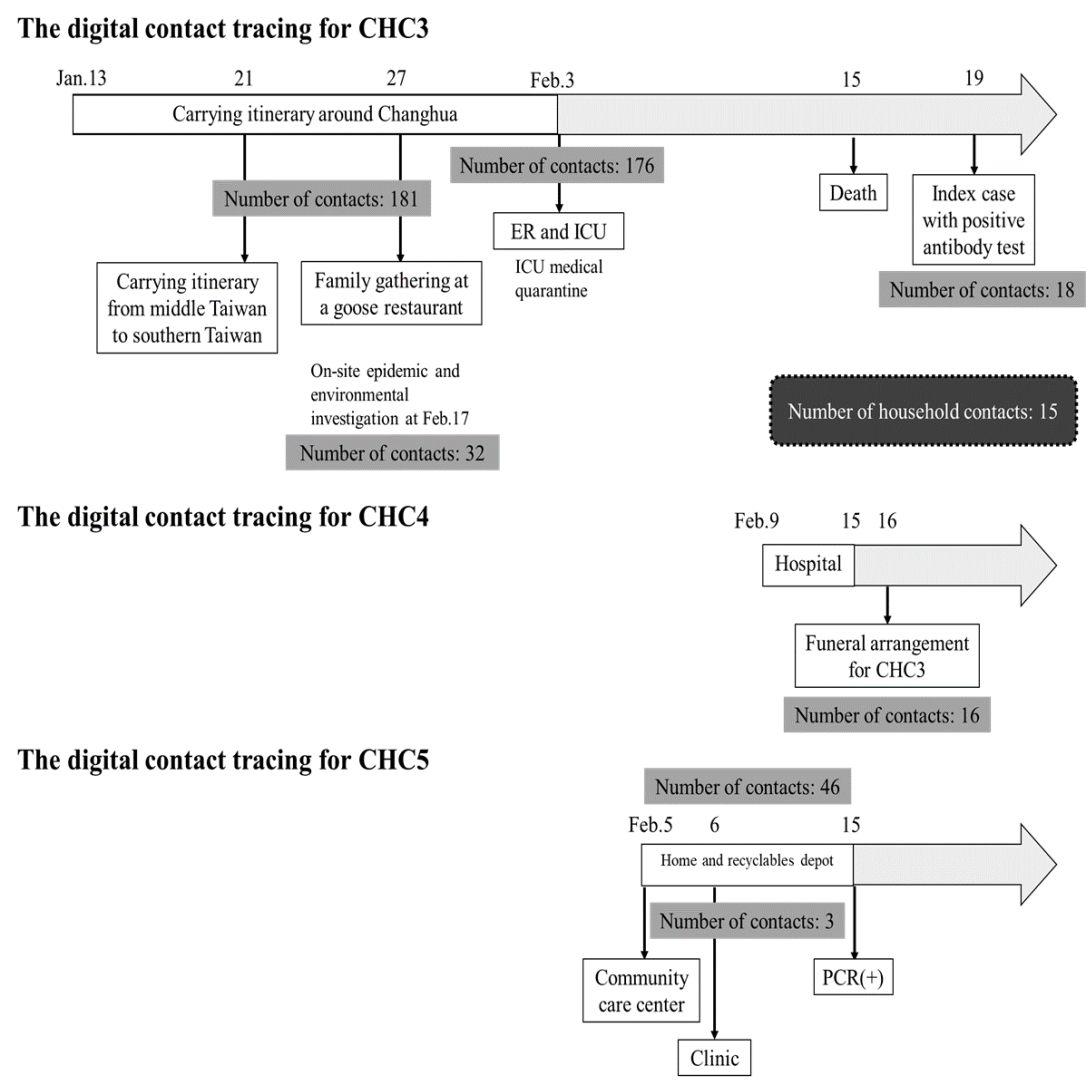
Step 3 of contact tracing of epidemic investigation in cluster 2—trace the history of contacts by using digital contact tracing system. CHC: case in Changhua; ER: emergency room; ICU: intensive care unit; PCR: polymerase chain reaction.

Further contact tracing for CHC3 identified 4 asymptomatic household members positive for COVID-19 (cases 4-7 in Changhua; Figure 5). As CHC3 had worked as a pak-pai taxi chauffeur during the transmission period, the Changhua County Public Health Bureau had to rely on both traditional and digital methods to conduct occupational contact tracing, involving the collection of anonymous data. With the assistance of integrated big data and digital contact tracing, the bureau was able to trace and identify >600 individuals who had occupational or social contacts with the cluster. The contact tracing process included several steps: clarifying CHC3’s family contact structure (Figure 5), identifying risk information using digitalized systems (Figure 6), and tracking the history of contacts using a digital contact tracing system (Figure 7). Following these steps, the pathways of contacts within households, workplaces, health care systems, and social occasions were elucidated, resulting in a total of 665 contacts traced within 30 days for this cluster (from January 13, 2020, to February 12, 2020). Table 1 summarizes the details about all the contacts traced in this clustered event and the results of RT-PCR tests, including the types and positive rates of cases in Changhua.

The estimated contact rate and overall positive rate were 22 per day (ie, 665/30, representing that 665 contacts took place during the 30-day period) and 0.6% (4/665), respectively. Assuming a 7-day infectious period [33,34], Rt was thus estimated as 0.92 (contact rate of 22 per day × transmission probability of 0.006 per contact × infective period of 7 days), indicating the successful containment of community-acquired outbreak for this clustered event.

**Table 1:** Number of contacts, results of polymerase chain reaction (PCR) testing, and results of the test according to contact categories in cluster 2 for each case in Changhua (CHC).

Index cases and categories of contacts and subtotal			Contacts (N=665), n (%)		PCR testing, n (%)^a^		Positive, n (%)^b^		Negative, n (%)^c^
**CHC0**									
	Social contacts	18 (2.7)		18 (100)		0 (0)		18 (100)	
**CHC3 and CHC4**									
	Household	15 (2.3)		15 (100)		4 (27)		11 (73)	
	Passengers	181 (27.2)		181 (100)		0 (0)		181 (100)	
	Hospitals	176 (26.5)		176 (100)		0 (0)		176 (100)	
	Primary clinic	25 (3.6)		25 (100)		0 (0)		25 (100)	
	Community pharmacy	5 (0.8)		5 (100)		0 (0)		5 (100)	
	Workplace	55 (8.3)		55 (100)		0 (0)		55 (100)	
	Restaurant	32 (4.8)		32 (100)		0 (0)		32 (100)	
	Social contacts	16 (2.4)		16 (100)		0 (0)		16 (100)	
	Subtotal	505 (75.9)		505 (100)		4 (0.8)		501 (99.2)	
**CHC5**									
	Social contacts	46 (6.9)		46 (100)		0 (0)		46 (100)	
	Primary clinic	3 (0.5)		3 (100)		0 (0)		3 (100)	
	Subtotal	49 (7.4)		49 (100)		0 (0)		49 (100)	
**CHC6**									
	Workplace	58 (8.7)		58 (100)		0 (0)		58 (100)	
	Primary clinic	19 (2.9)		19 (100)		0 (0)		19 (100)	
	Restaurant	2 (0.3)		2 (100)		0 (0)		2 (100)	
	Subtotal	79 (11.9)		79 (100)		0 (0)		79 (100)	
**CHC7**									
	Primary clinic	6 (0.9)		6 (100)		0 (0)		6 (100)	
	Workplace	8 (1.2)		8 (100)		0 (0)		8 (100)	
	Subtotal	14 (2.1)		14 (100)		0 (0)		14 (100)	

^a^PCR testing (total): 665/665, 100%.

^b^Positive (total): 4/665, 0.6%.

^c^Negative (total): 661/665, 99.4%.

### Cluster Investigation 3 (Foreign Worker Cluster With Extensive Contact History)

On July 29, 2020, a Belgian engineer working in Changhua, Taiwan, tested positive for COVID-19 during his routine RT-PCR test before an international flight (reported as case number 469 by the Central Epidemic Command Center in Taiwan). Case 469 had arrived in Taiwan on May 1, 2020, and had been in the country for 3 months (Figure S2C in Multimedia Appendix 1). Therefore, the patient was classified as a domestic case with a potential exposure period to infection from January 25, 2020, to July 8, 2020. The infectious period was estimated to be from July 9 to 29, 2020 (Figure S2C in Multimedia Appendix 1).

Initial investigations revealed that case 469 had not consistently worn a mask in public areas and had a sociable lifestyle. Figure S2C in Multimedia Appendix 1 displays the timeline of this case, including specific exposure instances. To prevent a community-acquired outbreak, the Changhua County Public Health Bureau immediately initiated contact tracing procedures, using digital contact tracing to collect information about contacts in hotels, workplaces, gyms, bars, and social activities related to case 469. A total of 462 potential contacts were identified, and the numbers and containment measures are summarized in Table 2. Of the 462 contacts, 47 (10.2%) were placed in self-isolation, and 415 (89.8%) were advised to practice self–health management. None of these contacts (0/462, 0%) tested positive for SARS-CoV-2 infection in RT-PCR tests.

**Table 2:** Number of contacts according to categories and their corresponding implementation of measures.

Categories of contacts	Contacts (n=462), n (%)	Measure, n (%)	
		Self-isolation^a^	Self–health management^b^
Workplace	66 (14.3)	26 (39)	40 (61)
Restaurant	4 (0.9)	0 (0)	4 (100)
Gym	246 (53.2)	0 (0)	246 (100)
Social contacts	146 (31.6)	21 (14.4)	125 (85.6)

^a^Total: 47/462, 10.2%.

^b^Total: 415/462, 89.8%.

## Discussion

### Main Findings

In this study, we have demonstrated the implementation of digital cluster investigation by using 3 empirical SARS-CoV-2 infection clusters in the early phase of the pandemic, when D614G was the dominant strain. However, the optimization of digital contact tracing requires updated information, varying with different VOCs from D614G and Alpha until Omicron and its subvariants to provide precise containment, including NPIs, antiviral therapy, and vaccination.

The implementation of digital contact tracing was made possible by integrating the public health, civil affairs, and police departments with the help of IT to collect detailed TOCC information at the individual level that could be retrieved using the NHI platform to achieve the goal of efficiently and precisely containing the COVID-19 outbreak in a community. The analysis focused on 3 empirical clusters of COVID-19 cases in Changhua, Taiwan, which included the first family cluster infection, the first community cluster infection in Taiwan, and a foreign worker cluster with an extensive history of contacts. These clusters served as examples of how digital contact tracing was effectively used for EID containment. Although there was no evidence of transmission within the household cluster and foreign worker cluster, the community cluster in Changhua (stemming from CHC3) had an estimated Rt of 0.92. This suggests confined transmission within the community following a prompt intervention aided by digital contact tracing.

### Evolution of Digital Contact Tracing in Taiwan

Following the successful experience in using digital contact tracing for containing COVID-19 among the large number of contacts (n=627,386) [5], a series of digital approaches was adopted. These included the incorporation of COVID-19 into the TRACE platform that has been established since 2017 [35]; development of a Bluetooth-based contact tracing platform, social distancing 2.0 [36]; a process of digital transformation for disease control [37]; and digital health governance during COVID-19 [38].

Since the onset of the COVID-19 pandemic in 2020, various frameworks and methods for digital contact tracing have been developed [8,10,11,13-23]. In addition to techniques aimed at enhancing the efficiency of identifying individuals at risk of infection owing to contact with pathogens, we have demonstrated a comprehensive strategy that integrates this information with traditional approaches. This strategy includes specific implementation steps for using big data analytics to curb the spread of COVID-19 within a community.

Despite the benefits of digital contact tracing in monitoring and preventing COVID-19 transmission, its implementation is still debatable because of ethical concerns [10,39,40]. The main issue lies in the violation of individual autonomy and privacy as a result of collecting the detailed information required for tracing the history of contact and assessing the risk of disease spread. These humanitarian aspects were emphasized during the implementation of digital contact tracing in the early phase of the COVID-19 pandemic. Ethical guidelines for contact tracing apps were proposed in 2020 [40]. These guidelines outline 4 major principles for collecting individual data: necessity; proportionality; sufficient effectiveness, timeliness, popularity, and accuracy; and temporariness. The digital contact tracing implemented in Taiwan fulfills all of the 4 principles. By using the well-established public service of the NHI, authorized professionals may assess various information, including travel, quarantine location, and health status, using a smart IC card for identity and media for data retrieval. The retention of tracing data for no more than 28 days ensures compliance with the principle of temporariness. These approaches guarantee both flexibility and security in digital contact tracing. Nevertheless, close monitoring for following these ethical guidelines has been implemented for all forms of digital contact tracing.

### The Implementation of Digital Contact Tracing for EID

The transition from traditional contact tracing to a digital strategy supported by big data analytics was developed using detailed information to assess the risk of disease spread, on the basis of which an effective containment measure could be applied; this serves as the first illustration of household clustered transmission. The system integrated big data and various digital contact tracing resources, offering a solid foundation for prompt action, which is crucial for stopping the spread of COVID-19 within a community.

In the second illustration of community cluster infection, collaboration between the public health sector and the police department enhanced the digital contact tracing, allowing 665 contacts to be traced and quarantined and further cases to be efficiently isolated. The likelihood of a community-acquired outbreak being contained is thanks to this improved digital contact tracing for the family and occupational contacts of the pak-pai taxi chauffeur, who covered Changhua county and Taichung city (a metropolitan area close to Changhua county, as illustrated in Figure S3 in Multimedia Appendix 1). For instance, case 7 in Changhua, a family member and also a contact of CHC3, who was later confirmed as a COVID-19 case, resides in Taichung and works there. Without the digital contact tracing system, it would have been impossible to precisely implement isolation and quarantine measures and quickly identify the contacts of case 7 in Changhua.

The final investigation focused on a series of incidents linked to a foreign engineer. The index case had an extensive network of contacts and had traveled to Changhua county, Taipei city, and Yilan county during his infectious period (Figure S2C in Multimedia Appendix 1). The digital contact tracing system enabled the reconstruction of potential contact pathways and timelines, facilitating local health authorities in promptly implementing containment measures. Owing to these precise containment measures supported by digital contact tracing, in addition to the widespread use of masks, monitoring for COVID-19 symptoms including body temperature, and social distancing measures, the risk of a large-scale community-acquired outbreak in Taiwan remains low.

These initial outbreaks in Changhua were contained to a small number of clusters that were manageable for Changhua’s health care infrastructure owing to the combination of digital contact tracing for targeted monitoring and surveillance and universal NPIs governed by a national level-3 alert [30,31]. The large-scale community outbreak caused by the Alpha VOC, which struck Taiwan between May 2021 and July 2021, was also mitigated by using this digital contact tracing method.

Incorporating the proposed digital contact tracing method into our public health strategy offers an efficient tool for managing community-acquired outbreaks. Moreover, the synergistic use of this digital approach alongside comprehensive databases holds significant promise in the timely identification of emerging variants or subvariants (such as XBB.1.5, which descends from Omicron BA.2) of EIDs. These variants may originate from imported cases or emerge within our domestic setting, akin to the scenario of Alpha and Omicron outbreak that occurred in Taiwan [31]. Drawing lessons from the COVID-19 pandemic, we recognize the importance of monitoring asymptomatic and presymptomatic imported cases upon their arrival in Taiwan. This can be efficiently achieved by harnessing immigration registries and implementing community surveillance mechanisms for tracking COVID-19 transmission. Integrating these data sources with our NHI database platform, which also encompasses communicable disease reporting systems, empowers us to facilitate early interventions. This proactive approach is instrumental in halting further transmission stemming from widespread community-acquired COVID-19 outbreaks and, ultimately, mitigating the risk of another EID pandemic. Without loss of generalizability, we used COVID-19 as an illustration for triggering the digital transformation of contact tracing. With the advent of digital contact tracing, timely decision-making can facilitate the containment and mitigation of EID.

In this study, we used Rt as an indicator to evaluate the effectiveness of contact tracing. However, in addition to digital contact tracing, there are many factors that affect Rt, such as the strategies for testing and isolating high-risk contacts, transmissibility and pathogenesis of VOCs, and accessibility of medical and public health resources. Evaluating the performance of digital contact tracing, using indexes such as the number of contacts required to identify a confirmed case, and performing sensitivity and specificity evaluations can help monitor the efficiency and adequacy of the system. These performance metrics also aid health policymakers in allocating limited resources, including those needed for isolation and testing, and the solicited indirect costs as a result of all warning messages.

In communities with a low risk of EID transmission, stringent criteria for determining the contacts eligible for NPIs are warranted, whereas the opposite holds true for areas experiencing outbreaks. These criteria are also influenced by the transmissibility and severity of an EID. Balancing the digital contact tracing system to address the trade-off between identifying all contacts to reduce the risk of missing individuals who are potentially infected and targeting a subgroup of contacts with high risk of infection is the objective of an ongoing study.

### Conclusions

In conclusion, we have demonstrated with the COVID-19 cluster investigation how the contact tracing process can be digitized by integrating multisectoral databases to provide the necessary information for risk assessment and decision-making in a timely manner. The better use of the contact tracing technique facilitates the efficient containment of community-acquired outbreaks and the prevention of subsequent large-scale outbreaks, especially when facing EIDs in the future.

## Appendix

Multimedia Appendix 1 Supplementary figures showing the digital contact tracing platform and the timelines and the geography of the evolution of COVID-19 cases in Changhua, Taiwan.

## Abbreviations

CHC: case in Changhua
EID: emerging infectious disease
NHI: National Health Insurance
NPI: nonpharmaceutical intervention
R0: basic reproduction number
Rt: effective reproduction number
RT-PCR: reverse transcriptase polymerase chain reaction
TOCC: travel, occupation, contact, and cluster
VOC: variant of concern
