# Supplementary material for: Community-Based Digital Contact Tracing of Emerging Infectious Diseases: Design and Implementation Study With Empirical COVID-19 Cases
Source: J Med Internet Res. 2023 Nov 8;25:e47219. doi: 10.2196/47219 (PMC10666017; doi:10.2196/47219)

**Supplementary Figures**

**Figure S1. Digitalized contact tracking platform**

**
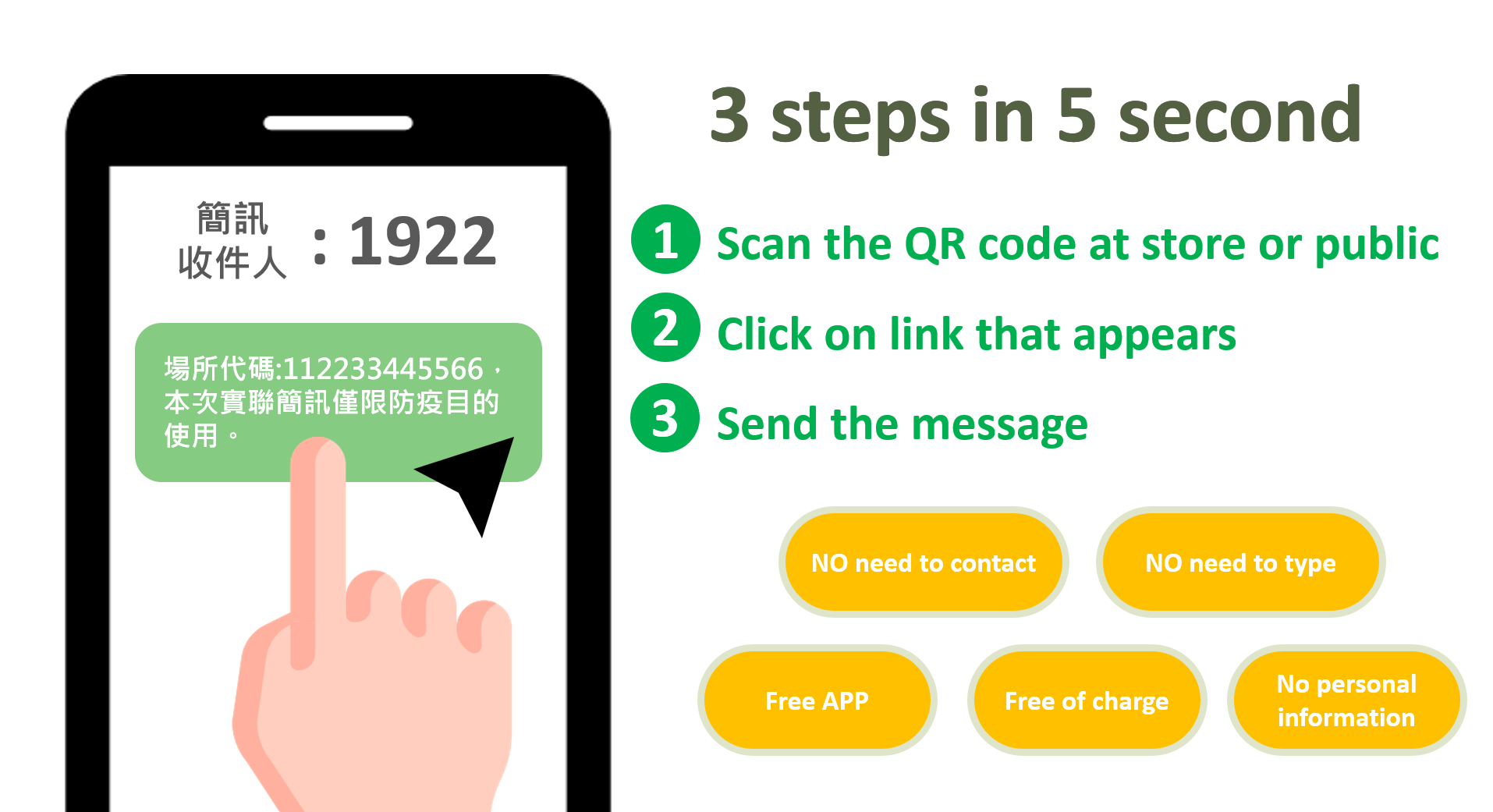
**

**Figure S2. The pathway of three examples in Changhua**

1. **Cluster Investigation 1 (First household infection in Taiwan)**


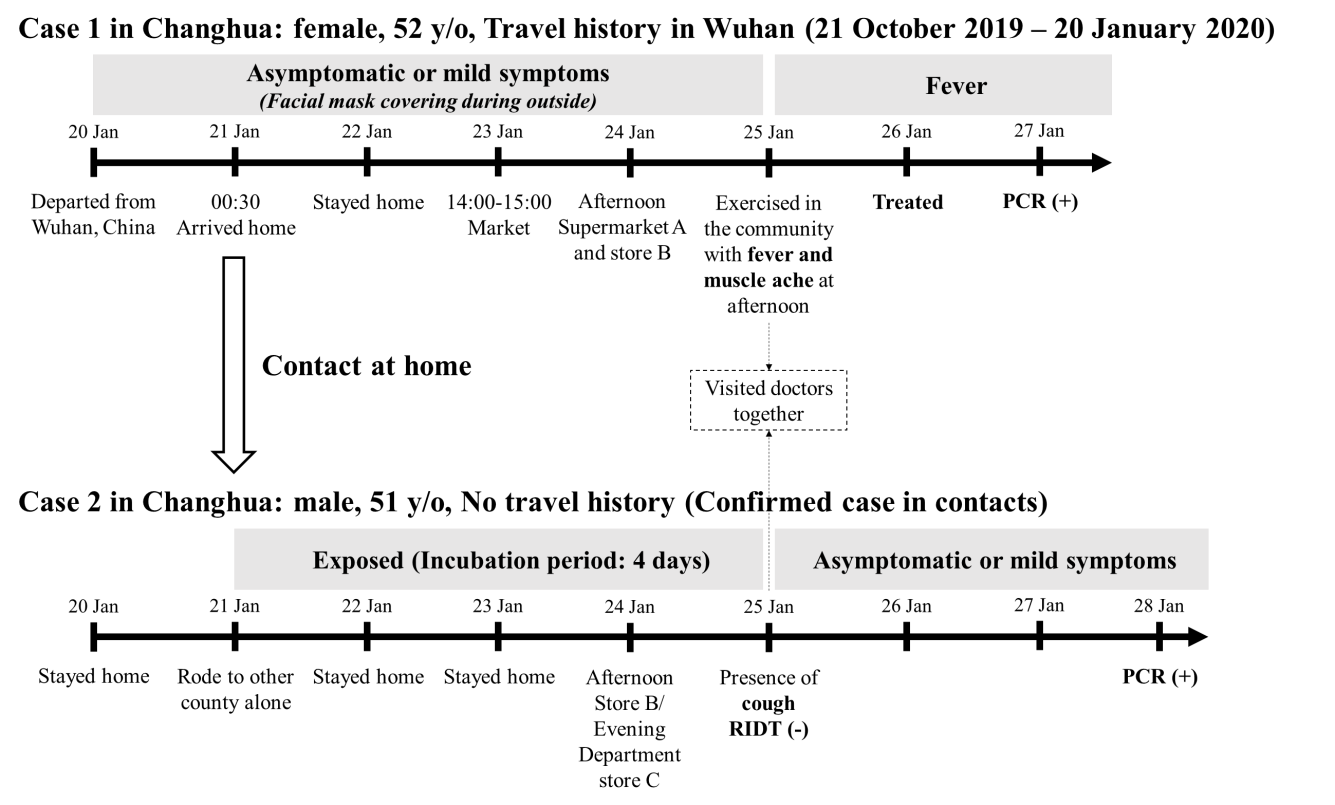


RIDT: Rapid influenza diagnostic test

1. **Cluster Investigation 2 (First community cluster infection in Taiwan)**


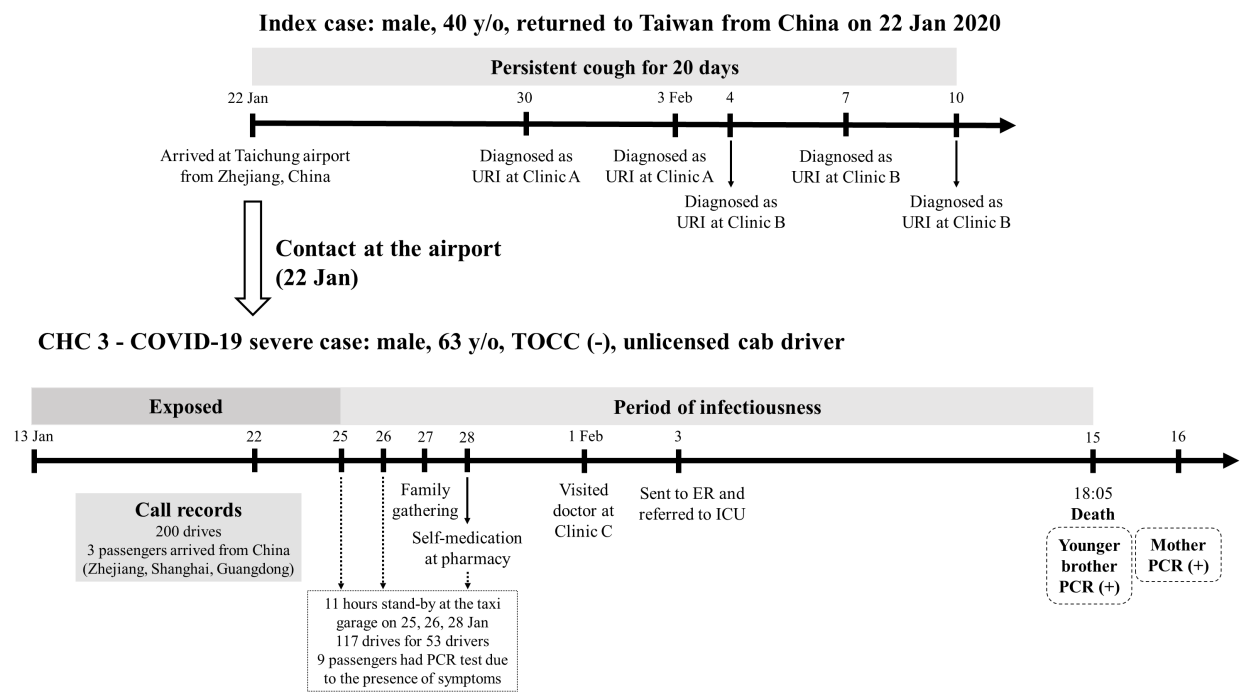


URI: upper respiratory infection; ER: emergency room; ICU: intensive care unit

1. **Cluster Investigation 3 (Foreign worker)**


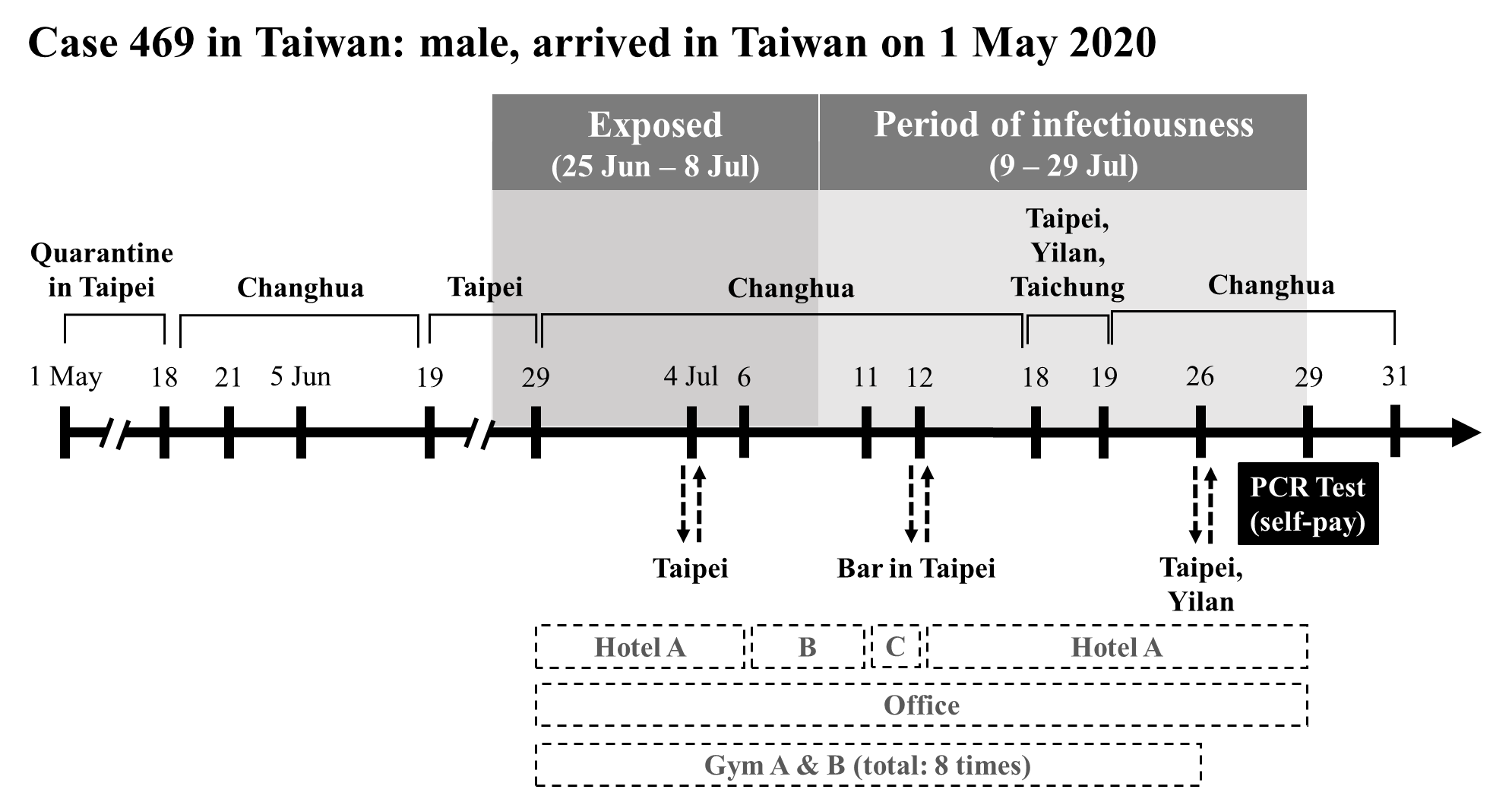


**Figure S3. The pathway of three examples in Changhua**


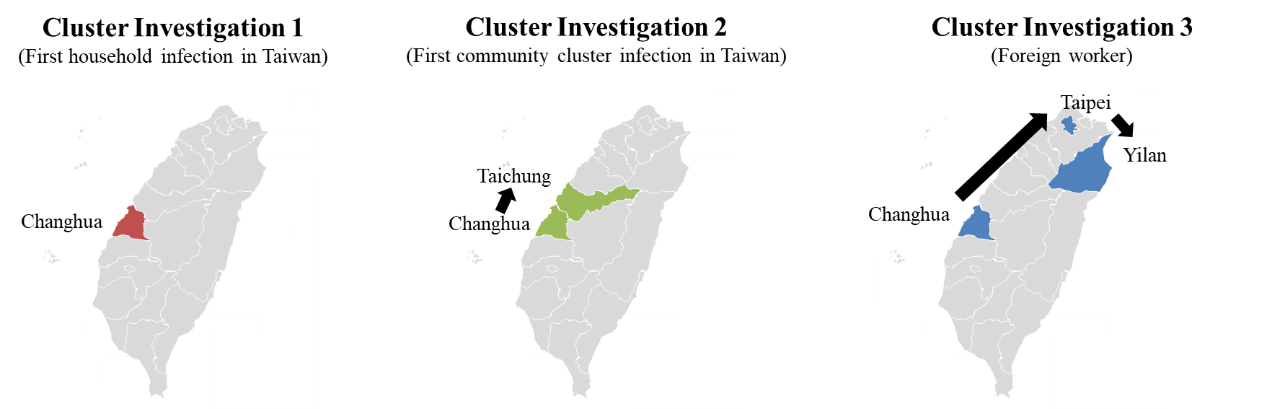

Supplement: Multimedia Appendix 1 [file jmir_v25i1e47219_app1.docx]
